# Arterial to jugular‐bulb lactate difference in patients undergoing elective brain tumor craniotomy

**DOI:** 10.14814/phy2.70084

**Published:** 2024-10-16

**Authors:** Alexandra Vassilieva, Markus Harboe Olsen, Jane Skjøth‐Rasmussen, Kirsten Møller, Martin Kryspin Sørensen

**Affiliations:** ^1^ Copenhagen Neuroanaesthesiology and Neurointensive Care Research Group (CONICA), Department of Neuroanaesthesiology, The Neuroscience Centre Copenhagen University Hospital – Rigshospitalet Copenhagen Denmark; ^2^ Department of Neurosurgery, The Neuroscience Centre Copenhagen University Hospital – Rigshospitalet Copenhagen Denmark; ^3^ Department of Clinical Medicine, Faculty of Health Sciences University of Copenhagen Copenhagen Denmark

**Keywords:** brain tumor, cancer, craniotomy, hyperlactatemia, lactate, neurosurgery

## Abstract

Hyperlactatemia is common during tumor craniotomy, but the underlying pathophysiology is unclear. This study measured simultaneous arterial and jugular‐bulb lactate concentrations in patients undergoing brain tumor craniotomy to investigate the hypothesis that hyperlactatemia was associated with a net cerebrovascular lactate input. In 20 patients, arterial and jugular‐bulb blood was collected hourly from the start of surgery to 6 h postoperatively for measurement of lactate, glucose, and oxygen concentration. For each marker, data were analyzed using a linear mixed‐effects model with jugular‐bulb concentration as dependent variable, arterial concentration as fixed effect, and patient as random effect. Furthermore, we generated regression lines between arterial and jugular‐bulb concentrations. The slope of the regression line between arterial and jugular‐bulb lactate was 0.95 (95% CI 0.93–0.97, *R* ^2^ = 0.98), indicating that increasing arterial lactate levels were associated with an increasingly positive net cerebrovascular balance (net input). The line crossed the identity line at 2.86 (95% CI 0.57–5.16) mmol/L, indicating that lower levels of lactate were associated with a negative net cerebrovascular balance (net output). This suggests a switch from net lactate output during normolactatemia towards net input during hyperlactatemia. Hyperlactatemia in tumor‐craniotomy patients probably does not originate from the brain.

## Graphical abstract

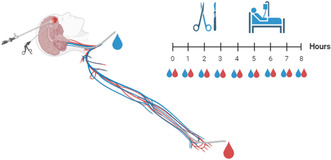

## 1 INTRODUCTION

Lactate is an intermediate metabolite produced during glycolysis and can accumulate as a result of a wide range of underlying conditions, including hypoxia, sepsis, liver dysfunction, heart failure, and certain medications (Adeva‐Andany et al., 2014). Lactate is widely recognized as an indicator of global or regional ischemia (Adeva‐Andany et al., 2014), but can also simply indicate increased glycolysis, for example, during increased sympathetic activity, and may also occur during hyperglycemia (Wisneski et al., 1990).

Hyperlactatemia (S‐lactate >2.0 mmol/L) occurs in up to 67% of patients undergoing tumor craniotomy (Brallier et al., 2017; Cata et al., 2017; de Smalen et al., 2020; Fazili et al., 2021; Ioannoni et al., 2020; Kohli‐Seth et al., 2011; Romano et al., 2019; Yoshikawa et al., 2018). The underlying mechanisms are unknown, but the risk varies depending on, for example, tumor type (Bharadwaj et al., 2015; Maldonado et al., 2019; Mariappan et al., 2015; Shih et al., 2017), prolonged duration of surgery (de Smalen et al., 2020), type of anesthesia (Bonhomme et al., 2009), and body mass index (Garavaglia et al., 2013).

In healthy humans during normolactatemia, there is a net efflux of lactate from the cerebrovascular compartment (van Hall et al., 2009); however, during hyperlactatemia in healthy volunteers, lactate is transported into and metabolized by the brain (Boumezbeur et al., 2010; van Hall et al., 2009). Furthermore, an exogenous lactate infusion reduces cerebral glucose uptake and metabolism at euglycemia (Boumezbeur et al., 2010; Smith et al., 2003; van Hall et al., 2009).

In patients with traumatic brain injury, a hypertonic sodium lactate infusion is associated with a switch from a lactate net output to a net input (Wolahan et al., 2018); it has been suggested that lactate may compensate for the decreased cerebral metabolic rate of glucose in these patients (Glenn et al., 2015).

The net cerebrovascular direction of lactate in patients undergoing tumor craniotomy is unknown. Therefore, in this study our aim was to measure arterial to jugular‐bulb difference (a‐jD) of lactate as a proxy for the net cerebrovascular flux of lactate in the perioperative phase in these patients. We hypothesized that hyperlactatemia was associated with a net cerebrovascular influx of lactate.

## 2 METHODS

This study was a prespecified substudy nested within a larger prospective study of hyperlactatemia during and after tumor craniotomy, which was approved by the Research Ethics Committee of the Capital Region (identifier: H‐20011650; 21 July 2020) and the Danish Data Protection Agency (identifier: P‐2020‐566; 18 May 2020). A detailed study protocol, which included the substudy reported here, has been published previously (Vassilieva et al., 2022) and was registered on clinicaltrials.gov (identifier: NCT04410315; 14 May 2020) before inclusion of the first participant. Patients were recruited from November 2021 to January 2022 during their preoperative assessment at the Department of Neuroanaesthesiology and the Department of Neurosurgery, Copenhagen University Hospital – Rigshospitalet, Denmark. Eligible patients were adults (≥18 years old) with a brain tumor, who were scheduled to undergo elective craniotomy (Table 1). Patients undergoing craniotomy for a stereotactic biopsy of a brain tumor or resection of pituitary tumors were excluded from the study. The study adhered to the Declaration of Helsinki. Oral and written informed consent was obtained from all study participants.

**TABLE 1:** Patient characteristics.

Number of study participants	20
Age (median [IQR])	63.0 [53.8, 69.0]
Sex (%)	
Male	11 (55)
Female	9 (45)
ASA score (%)	
1	1 (5)
2	11 (55)
3	9 (45)
BMI (median [IQR])	25.2 [24.0, 27.8]
Charlson Score (median [IQR])	4.0 [3.0, 5.2]
Diabetes (%)	
Yes	1 (5)
None or diet‐controlled	19 (95)
Liver disease (%)	
No	20 (100)
Kidney disease (%)	
No	20 (100)
Tumor type (%)	
Meningioma grade 1	9 (45)
Meningioma grade 2	1 (5)
Cavernous hemangioma	2 (10)
Glioblastoma	6 (30)
Lung cancer metastasis	1 (5)
Malignant melanoma metastasis	1 (5)
Preoperative prednisolone (mg) (median [IQR])	0.0 [0.0, 50.0]
Intraoperative dexamethasone (mg) (median [IQR])	24.0 [6.0, 24.0]
Surgery, duration (hours) (median [IQR])	1.6 [1.2, 2.2]
Positioning (%)	
Prone	4 (20)
Supine	16 (80)
Resection (%)	
Macro total	16 (80)
Incomplete	4 (20)
Anesthesia duration in hours (median [IQR])	3.0 [2.5, 3.4]
Hypotension (%)	
No	18 (90)
MAP <60 mmHg for >15 min	
Yes	2 (10)
Metaoxedrine, total dose (mg) (median [IQR])	0.2 [0.2, 0.2]
Ephedrine, total dose (mg) (median [IQR])	15.0 [10.0, 20.0]
Noradrenaline, total dose (ug) (median [IQR])	476.0 [256.5, 654.8]
Preoperative hemoglobin (mM) (median [IQR])	7.8 [6.9, 8.4]
Blood loss (mL) (median [IQR])^a^	350.0 [237.5, 600.0]

*Note*: Values are median [5%–95% range] or *n* (%).

Abbreviations: ASA, American Society of Anesthesiologists; BMI, body mass index; IQR, interquartile range; MAP, mean arterial pressure.

^a^ One patient received a transfusion with 250 mL of red blood cell suspension (SAG‐M) during surgery.

### 2.1 Standard treatment

All patients received general anesthesia, with an induction dose of 1–2 mg/kg of propofol, followed by continuous infusion of propofol (10 mg/mL) and remifentanil (50 μg/mL). All patients underwent endotracheal intubation. An arterial catheter was placed in the radial artery as part of the perioperative monitoring.

Dexamethasone was given after anesthesia induction to all patients. Prophylactic intravenous antibiotics were administered pre‐incision. Mean arterial pressure was kept ≥80 mmHg by continuous vasopressor infusion (noradrenaline 10 μg/mL) or bolus injections of ephedrine (50 mg/mL) or metaoxedrine (1 mg/mL). During surgery, patients received 4 mg of ondansetrone, 0.05 mg/kg of morphine, and 1000 mg of paracetamol intravenously.

Postoperatively, patients were extubated while still in the operating theater and transported to the Post Anaesthesia Care Unit (PACU) for at least 6 h' monitoring. Postoperative pain was managed with 400 mg of celecoxib and 1000 mg of paracetamol at fixed intervals and with morphine titrated until effect. Nausea or vomiting were treated with ondansetrone, dehydrobenzperidol, and/or antihistamine.

### 2.2 Intervention and data collection

Jugular‐bulb catheterization is generally considered a simple and safe procedure with a low incidence of serious complications (Gemma et al., 1998). After anesthesia induction, a single‐lumen central venous catheter (Arrow 16 G, 16 cm, Teleflex Inc., Wayne, Pennsylvania) was placed with the tip in the jugular‐bulb using ultrasound guidance.

Blood samples were drawn simultaneously from the jugular‐bulb catheter and the arterial catheter at the time of surgical incision and hourly thereafter, until patients had spent 6 h in the PACU. Medical history, treatment, and perioperative monitoring data were collected from the patient journal.

### 2.3 Blood sample analysis and calculations

The paired blood samples were analyzed immediately using a blood‐gas analyzer (ABL800 flex, Radiometer, Denmark). Arterial blood samples were obtained using the same cannula utilized for invasive blood pressure monitoring, which is equipped with continuous slow flush of sterile solution to prevent clotting.

Glucose, lactate, and hemoglobin (Hb) were measured in mmol/L. Hyperlactatemia was defined as a maximum serum lactate >2.0 mmol/L (Khosravani et al., 2009). SaO_2_ and SvO_2_ were measured as the oxyhemoglobin fraction of hemoglobin in arterial and jugular‐bulb blood respectively. PaCO_2_, PaO_2_, and PjvO_2_ were measured in kPa (at 37°C and STPD). The arterial (Ca) and jugular‐bulb (Cjv) content of oxygen (CaO_2_ and CjvO_2_, respectively) in mmol/L was calculated as follows:

$${\mathrm{CaO}}_{2}=\left(\mathrm{OBC}\times {\mathrm{SaO}}_{2}\times \mathrm{Hb}\right)+\left(\alpha \times {\mathrm{PaO}}_{2}\right)$$

$${\text{CjvO}}_{2}=\left(\mathrm{OBC}\times {\text{SjvO}}_{2}\times \mathrm{Hb}\right)+\left(\alpha \times {\text{PjvO}}_{2}\right),$$

where OBC is the oxygen binding capacity of hemoglobin (=0.96 mmol O_2_ per mmol Hb) and α is the solubility of oxygen in plasma (=0.01 mmol/L/kPa) (Møller et al., 2002).

The arterial to jugular‐bulb difference (a‐jD) of substance x was calculated as

$$a-\mathrm{jD}=\mathrm{Ca}\left(x\right)-\mathrm{Cjv}\left(x\right)$$

Accordingly (and by convention), a cerebrovascular net *in*flux of a substance means that the a‐jD is *positive*, whereas a cerebrovascular net *ef*flux means that the a‐jD is *negative*.

As a supplementary analysis, to assess potential discrepancies between arterial and jugular‐bulb samples arising from dilution, measured concentrations of lactate, glucose, and oxygen content were adjusted based on the relative difference (in per cent) in hemoglobin between the simultaneous arterial and jugular‐bulb samples.

### 2.4 Statistical analyses

As little is known about the standard deviation of our continuous outcome measurements (the a‐jD of lactate, glucose, and oxygen content over time), it was not possible to calculate a sample size. Recommendations for a minimal sample size for a pilot study range from 12 (Julious, 2005) and upwards. For this observational physiological study, a sample size of 20 participants was chosen, as it was feasible and accounted for the potential risk and discomfort that jugular‐bulb catheterization might inflict on the study participants.

The relationship between jugular‐bulb (dependent variable) and arterial (independent variable) measurements of lactate, glucose, and oxygen content are presented in scatter plots with linear regression. A linear mixed‐effects model with arterial measurements as fixed effects and patients as random effects was generated to account for the repeated measurements over time.

For goodness‐of‐fit, *R* ^2^ was used to indicate how well the arterial variables explained the venous variables. No outliers were removed from the analyses. We used R version 4.3.1 (R Core Team, Vienna, Austria) for descriptive statistics and statistical analyses. The *ggplot2*‐package (version 3.4.4) was used for figures; the *tableone*‐package (version 0.13.2) for tables; and the *nlme*‐package (version 3.1‐164) was used for the linear mixed‐effects model.

## 3 RESULTS

**FIGURE 1:**
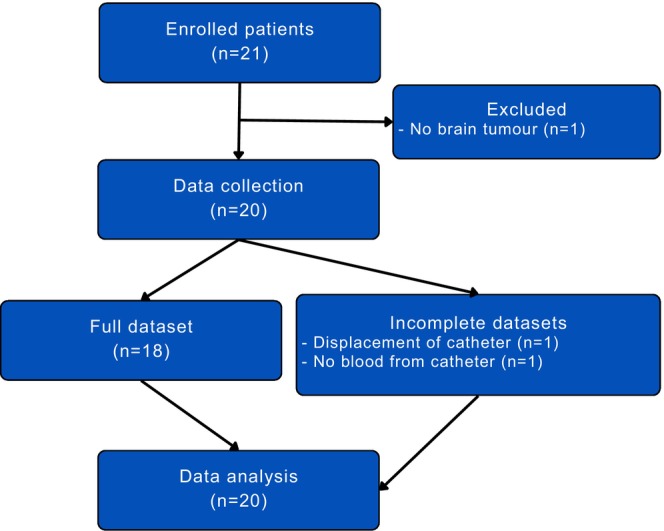
Participant flow diagram. Twenty‐one patients initially consented to participate in the study. One patient was excluded due to the absence of a brain tumor. All 20 patients were included in the data analysis, although two patients had incomplete datasets.

Twenty‐one patients gave oral and written informed consent to the study. Of the initial 20 patients who were enrolled, one was excluded because their intracranial pathology did not meet the inclusion criteria; an additional patient was included to reach the predetermined total of 20 patients for data analysis. We collected a full dataset from 18 of the 20 patients (Figure 1). In one case, blood withdrawal from the jugular‐bulb catheter ceased midway through the observational period, probably due to a postsurgical thrombogenic condition. In another, the catheter was displaced when the patient was turned from prone to supine position after surgery. All available data were included in the data analysis. In total, 150 paired arterial and jugular‐bulb blood samples were analyzed. SjvO_2_ ranged between 40% and 78%, consistent with a correct location of the jugular‐bulb catheter.

The median age was 63 years, 11 of the patients were male and the most common tumor type was meningioma (Table 1).

**FIGURE 2:**
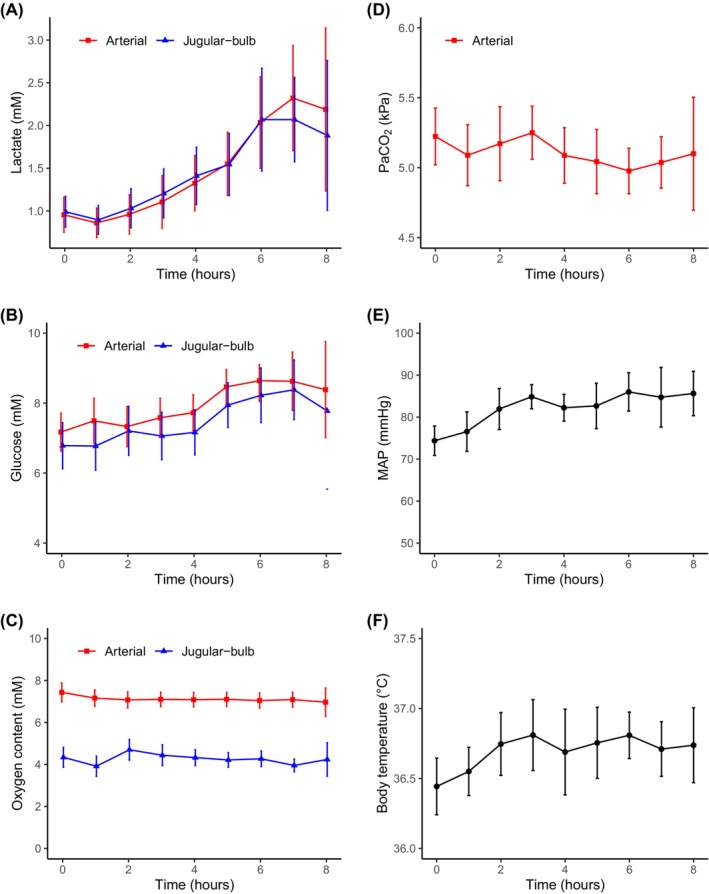
The time‐course of (a) lactate, (b) glucose, and (c) oxygen concentration, (d) arterial carbon dioxide tension (PaCO_2_), (e) mean arterial pressure (MAP), and (f) body temperature for all patients. Values are mean and 95% confidence intervals. Arterial measurements in red, jugular‐bulb measurements in blue, and MAP and body temperature measurements in black.

The mean arterial pressure, arterial pressure of carbon dioxide, and body temperature remained constant throughout the study (Figure 2). Hyperlactatemia (defined as at least one measurement of arterial lactate >2.0 mmol/L) was observed in 12 patients. Arterial and venous levels of both lactate and glucose gradually increased over the course of the study (Figure 2).

**FIGURE 3:**
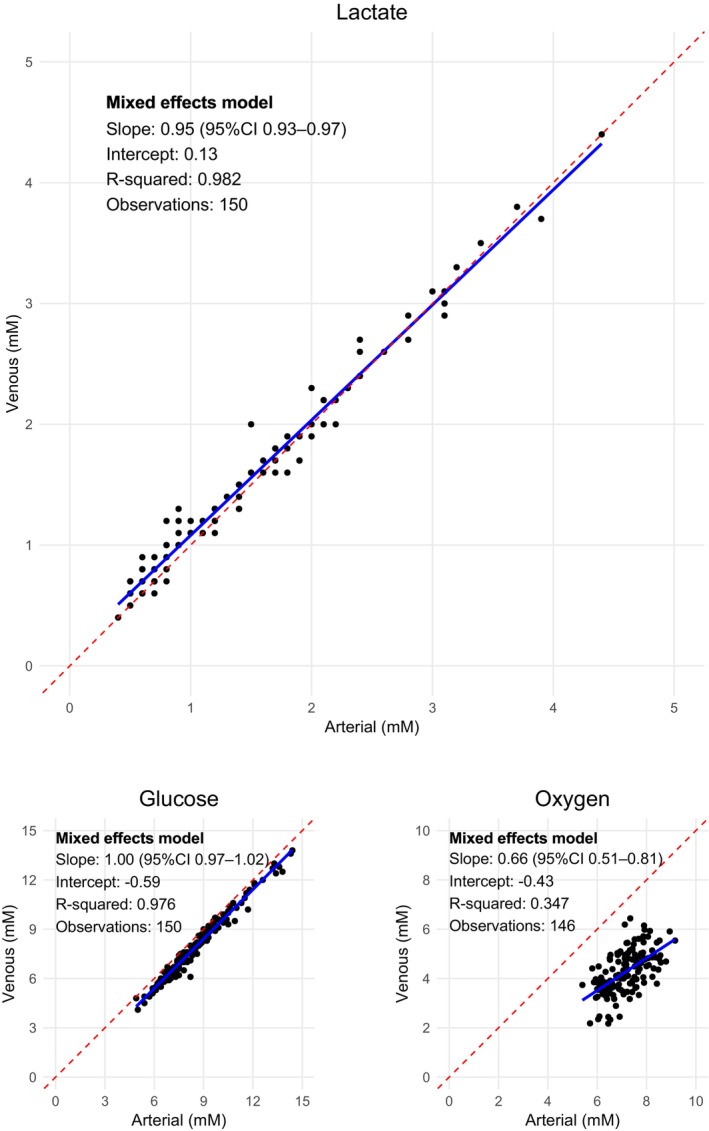
Linear mixed‐effects model of arterial to jugular‐bulb lactate, glucose, and oxygen content. Each point corresponds to a unique patient at a specific timepoint. The stippled red line is the identity line, with a slope of one. The full blue line is the linear regression line. The table in the upper left corner of each graph summarizes the results of a linear mixed‐effects model, where jugular‐bulb values are the outcome variable, arterial values are considered as fixed effects and individual patients are treated as random effects.

The mixed‐effects model showed a high goodness of fit for lactate and glucose with *R* ^2^ of 0.98 for both (Figure 3). The regression coefficient for jugular‐bulb to arterial lactate was 0.95 (95% CI 0.93–0.97), indicating a progressively lower level of jugular‐bulb lactate at higher arterial lactate levels. In other words, according to the model, at a normal arterial lactate levels at 1.00 mmol/L, the corresponding jugular‐bulb lactate level would be higher (1.08 mmol/L), indicating a net output; however, during hyperlactatemia at 5.0 mmol/L, the jugular‐bulb lactate level would be lower (4.91 mmol/L), indicating a net input. According to our model, the transition from output to input occurred at an arterial lactate level of ≥2.86 (95% CI 0.57–5.16) mmol/L.

The linear mixed‐effects models were not significantly altered by adjusting for concentrations of lactate, glucose, and oxygen content according to differences in hemoglobin between arterial and jugular‐bulb samples (Appendix S1).

## 4 DISCUSSION

This study explored the dynamics of arterial and jugular‐bulb lactate, glucose, and oxygen content during and after tumor craniotomy. We observed a change from a net cerebrovascular output towards a net input at progressively higher arterial lactate levels. By comparison, our analyses did not reveal a change in arterial to jugular‐bulb glucose difference with increasing glucose levels, indicating that hyperglycemia did not affect the cerebrovascular net input of glucose in these patients. Finally, the relationship between arterial and jugular‐bulb oxygen content was consistent with a progressive increase in arterial to jugular‐bulb oxygen difference when arterial oxygen content increased, which can be ascribed to the well‐known effect of arterial oxygen content on global cerebral blood flow (Hoiland et al., 2016; Paulson et al., 1973).

Our findings indicate a shift from cerebral lactate influx to efflux at an arterial lactate level of 2.86 mM. This is consistent with the observation that healthy volunteers showed an efflux at approximately 0.9 mM and an influx at ~3.9 and 6.9 mM (van Hall et al., 2009), whereas patients with severe traumatic brain injury, who also showed an efflux at ~0.9 mM, experienced an influx already during lactate infusion at levels of ~1.8 mM (Wolahan et al., 2018). The participants of these studies differed with respect to both the underlying condition (healthy vs. traumatic brain injury vs. intracranial tumor) and the origin of hyperlactatemia (physical activity combined with lactate infusion vs. isolated lactate infusion vs. perioperative). Notably, high‐intensity exercise in healthy humans, which has been reported to elevate lactate levels to 15–25 mM (Goodwin et al., 2007), that is, well above the threshold of 2.86 mM found here, also leads to increased cerebral lactate uptake (Rasmussen et al., 2011). Together, this indicates that hyperlactatemia‐associated lactate uptake occurs during a wide range of conditions, both in the healthy human brain as well as during severe global and focal brain injury, and that lactate may be an important alternative energy source to glucose in the brain at least during some of these conditions.

The cause of hyperlactatemia during brain tumor craniotomy is currently unknown. In this study, increased lactate levels were observed alongside elevated glucose levels without the presence of ischemia. This suggests that hyperlactatemia could be driven by hyperglycemia and the associated hyperglycolysis. A possible explanation, which requires further investigation, could be an interaction between glucocorticoid‐induced insulin resistance and the surgical stress response; notably, many of these patients are treated with glucocorticoids, both between diagnosis and surgery to reduce oedema and intraoperatively to diminish the risk of postsurgical hemorrhage.

The transport of lactate across the intact blood–brain barrier is facilitated by monocarboxylate transporters and occurs along lactate as well as H+ gradients (Hertz & Dienel, 2005); thus it is tempting to suggest that there is a relationship between brain interstitial and blood levels of H+ on one side and the flux of lactate on the other side. However, as we did not measure brain interstitial concentrations, we are unable to shed further light on this issue.

As tumors metabolize glucose to produce lactate, a phenomenon known as the Warburg effect (Warburg et al., 1927), the presence of a brain tumor could theoretically lead to release of lactate from the brain. This, however, should lead to an observation of a net *output* from the brain in conjunction with hyperlactatemia. The results of the present study suggest a net *input* of lactate during hyperlactatemia, in conjunction with a quite minimal net output during normolactatemia, which speaks against the brain as a major source of hyperlactatemia.

The major strength of this study was its inclusion of consecutive patients undergoing brain tumor craniotomy. It was rigorously conducted according to a prespecified protocol as part of the first prospective, observational study of hyperlactatemia in such patients.

The main limitation of this study is that cerebral perfusion was not directly measured, primarily because it was unfeasible in the clinical workflow. Thus, the absolute flux of lactate cannot be inferred from the present study. However, the observation of a shift in the net direction of lactate from an output during low blood lactate levels to an uptake during high levels does not depend on the measurement of cerebral blood flow.

We did not confirm the placement of the catheter tip with X‐ray imaging of the neck. However, ultrasound‐guided retrograde catheterization of the internal jugular vein is an easy procedure with a high success rate. Extracerebral contamination is minimal even when the catheter tip is placed a few centimeters from the base of the skull, which suggests a substantial margin of error for precise catheter placement (Jakobsen & Enevoldsen, 1989). Moreover, extracranial contamination is usually associated with high SjvO_2_ values; the values in this study were between 40% and 78%, consistent with a correct location of the catheter tip in the jugular‐bulb.

A growing body of evidence suggests that lactate directly contributes to brain energy metabolism, plays a central role in certain brain functions, and appears to be neuroprotective after brain injury (Brooks, 2020; Li et al., 2022). Others have suggested that tumor cells thrive on lactate as this molecule accommodates their high energy demand and is involved in angiogenesis, immune escape, and cell migration (San‐Millán & Brooks, 2017). Many brain tumors, especially gliomas, are infiltrative in nature and therefore difficult to remove in toto during surgery (Sanai & Berger, 2018). The remaining cancer cells could therefore have a survival advantage if subjected to hyperlactatemia after surgery, which can shorten the period of cancer remission. Considering the role of lactate in brain metabolism and the observed input during hyperlactatemia, further investigation into cancer recurrence in patients with hyperlactatemia during tumor craniotomy could be of clinical interest.

In conclusion, we found indications of a net lactate input during hyperlactatemia in patients undergoing craniotomy for brain tumor surgery. The finding suggests that the brain is not the source of hyperlactatemia in these patients.

## AUTHOR CONTRIBUTIONS

AV, JSR, KM, and MKS designed the study. AV conducted the study and collected data. AV and MHO analyzed the data. AV and KM wrote the initial draft. All authors critically revised and approved the final version of the manuscript, agreeing to be accountable for all aspects of the work. All persons designated as authors qualify for authorship, and all those who qualify for authorship are listed.

## FUNDING INFORMATION

Funded by “Oberstinde Kirsten Jensa la Cours legat”, “Hakon August Valdbjørn, Else Valdbjørn og Hugo Valdbjørns Fond”, “Jens og Maren Thestrups Legat til kræftforskning”, “Læge Agnethe Løvgreens Fond”, and The Novo Nordisk Foundation (ref. NNF20OC0065750).

## CONFLICT OF INTEREST STATEMENT

None of the remaining authors have any relevant non‐financial interest to declare.

## Supporting information


Appendix S1.

## Data Availability

All original “raw” data from which figures and tables summary data is generated is archived and fully available to the journal upon reasonable request.
