# Supplementary material for: Arterial to jugular‐bulb lactate difference in patients undergoing elective brain tumor craniotomy
Source: Physiol Rep. 2024 Oct 16;12(20):e70084. doi: 10.14814/phy2.70084 (PMC11483513; doi:10.14814/phy2.70084)
Supplement: Supplementary file 1 — Appendix S1. [file PHY2-12-e70084-s001.docx]

# Supporting information

# S1: Linear mixed-effects model for lactate, glucose, and oxygen content corrected for haemoglobin. Linear mixed-effects model of jugular-bulb values with arterial values as fixed effect and patient as a random factor. To correct for potential dilution-related differences between arterial and jugular-bulb concentrations, all concentrations were adjusted for haemoglobin levels. The calculated coefficients (intercept and slope) are shown with 95% confidence intervals and p-values.

|  | **Jugular-bulb lactate** | | |  |  |
| --- | --- | --- | --- | --- | --- |
| *Predictors* | | *Estimates* | *CI* |  |  |
| (Intercept) | | 0.12 | 0.09 – 0.16 |  | |
| Arterial lactate | | 0.96 | 0.94 – 0.98 |  | |
| Observations | | 150 | |  |  |
| R^2^ | | 0.986 | |  |  |
|  | **Jugular-bulb glucose** | | |  |  |
| *Predictors* | | *Estimates* | *CI* |  |  |
| (Intercept) | | -0.54 | -0.77 – -0.31 |  | |
| Arterial glucose | | 0.99 | 0.97 – 1.02 |  | |
| Observations | | 150 | |  |  |
| R^2^ | | 0.974 | |  |  |
|  | **Jugular-bulb oxygen content** | | |  |  |
| *Predictors* | | *Estimates* | *CI* |  |  |
| (Intercept) | | -0.68 | -1.73 – 0.38 |  | |
| Arterial oxygen content | | 0.69 | 0.55 – 0.84 |  | |
| Observations | | 146 | |  |  |
| R^2^ | | 0.373 | |  |  |
